# Molecular determinants of neoadjuvant chemotherapy resistance in breast cancer: An analysis of gene expression and tumor microenvironment

**DOI:** 10.1371/journal.pone.0334335

**Published:** 2025-10-14

**Authors:** Hedda Michelle Guevara-Nieto, Carlos A. Orozco-Castaño, Rafael Parra-Medina, Jenny Nathaly Poveda-Garavito, Jone Garai, Jovanny Zabaleta, Liliana López-Kleine, Alba Lucia Combita

**Affiliations:** 1 Grupo de Investigación en Biología del Cáncer, Instituto Nacional de Cancerología, Bogotá, Colombia; 2 Grupo de Investigación Traslacional en Oncología, Instituto Nacional de Cancerología, Bogotá, Colombia; 3 Doctorado en Oncología, Departamento de Patología, Facultad de Medicina, Universidad Nacional de Colombia, Bogotá, Colombia; 4 Departamento de Patología, Instituto Nacional de Cancerología, Bogotá, Colombia; 5 Departamento de Patología, Fundación Universitaria de Ciencias de la Salud, Bogotá, Colombia; 6 Stanley S. Scott Cancer Center, Louisiana State University Health Science Center (LSUHSC), New Orleans, Louisiana, United States of America; 7 Department of Interdisciplinary Oncology, Louisiana State University Health Science Center (LSUHSC), New Orleans, Louisiana, United States of America; 8 Departmento de Estadistica, Facultad de Ciencias, Universidad Nacional de Colombia, Bogotá, Colombia; 9 Departmento de Microbiología, Facultad de Medicina, Universidad Nacional de Colombia, Bogotá, Colombia; Weill Cornell University, UNITED STATES OF AMERICA

## Abstract

Neoadjuvant chemotherapy (NAC) is a critical component of breast cancer treatment, but the molecular mechanisms underlying resistance remain poorly understood. This study aimed to identify transcriptomic changes associated with NAC resistance across four breast cancer subtypes: Luminal A, Luminal B/HER2-positive, Luminal B/HER2-negative, and Triple-Negative Breast Cancer (TNBC). RNA-seq analysis was performed on paired pre- and post-NAC breast cancer samples from 32 non-responders. Differentially expressed genes (DEGs) were identified, and functional enrichment analyses were conducted. Protein-protein interaction (PPI) networks were constructed to identify hub genes. Tumor microenvironment (TME) infiltration was estimated using deconvolution algorithms. The results revealed distinct gene expression profiles between pre- and post-NAC samples, with *FOS* and *NR4A1* being common DEGs across all subtypes. Enriched pathways varied among subtypes, including signal transduction, estrogen biosynthesis, extracellular matrix organization, dendritic cell activation, and B cell activation. TME analysis showed increased infiltration of specific immune cell populations after NAC, including CD4 memory T cells, regulatory T cells, neutrophils, macrophages, and mast cells, varying by subtype. These findings suggest that NAC modulates gene expression, cellular activity, and TME interactions, potentially contributing to treatment resistance. Understanding the molecular determinants of NAC resistance is crucial for developing targeted therapeutic strategies and improving outcomes for breast cancer patients.

## Introduction

Neoadjuvant chemotherapy (NAC) is a cornerstone of primary breast cancer (BC) treatment. improving surgery outcomes and providing real-time efficacy assessment while offering overall survival (OS) and disease-free survival (DFS) rates comparable to adjuvant therapy. This equivalence allows clinicians to opt for NAC without compromising long-term outcomes [1,2]. Despite its benefits, NAC resistance remains a challenge. Tailored to tumor subtypes, NAC efficacy varies, sometimes reducing tumor size but increasing local recurrence risk [3,4].

Treatment resistance hinders long-term remission as resistant cancer subclones emerge during therapy [5]. Residual tumors often differ from pretreatment ones, with genetic and molecular changes driving resistance. NAC may not fully eliminate cancer but instead select resistant clones and promote metastasis by inducing cellular stress and remodeling the tumor microenvironment (TME) [6–8]. Understanding transcriptomic and molecular alterations in residual disease is crucial to overcoming resistance.

Similar challenges arise in esophageal adenocarcinoma (EAC), where NAC or chemoradiotherapy precedes surgery. Therapy response varies and predicts survival and recurrence. Some tumors are intrinsically resistant, while others mix sensitive and resistant cells [9]. In BC and EAC, most tumors likely harbor resistant subpopulations due to genetic or epigenetic changes, though these alterations are rare and hard to detect pre-treatment [9]. Beyond mutations, factors like hypoxia, cancer stem cell (CSC) phenotypes, and the stromal microenvironment further reduce therapeutic efficacy [10].

Tumor heterogeneity plays a central role in cancer progression and therapy resistance. Studies show tumors exhibit high cellular diversity, driving evolution of drug-resistant clones [10]. NAC resistance may arise from therapy-induced selection of resistant subpopulations, leading to increased cellular plasticity. Resistant cancer cells adapt their transcriptomic signature and interactome, acquiring phenotypes independent of drug-targeted pathways [11]. Genetic mutations contribute to intrinsic resistance, with alterations in key genes like *PIK3CA* promoting cell survival and proliferation, reducing chemotherapy effectiveness [12]. Cancer stem cells (CSCs) contribute to resistance by repopulating tumors after treatment, exhibiting mechanisms less responsive to standard therapies [12,13]. TME influences resistance through interactions with cancer cells, hypoxia, low pH, and stromal cell-mediated support, diminishing chemotherapy effectiveness [14,15]. Understanding genes, pathways, and molecular interactions involved in drug resistance is crucial for developing precision medicine strategies [16].

A promising approach to overcome NAC resistance involves identifying key genes contributing to resistance through compensatory pathways and protein interaction network alterations. Bioinformatics and systems biology enable comparative analysis of gene expression profiles between NAC-sensitive and NAC-resistant tumors, identifying molecular drivers of resistance. Moreover, hub genes involved in transcriptomic reprogramming and therapy resistance may serve as critical biomarkers for treatment response [17].

Hispanics/Latinos exhibit unique cancer incidence and mortality patterns compared to non-Hispanic Whites (NHW). Hispanic/Latino patients tend to be diagnosed at more advanced stages for several cancers, including breast, colorectal, prostate, and lung cancers [18]. Interestingly, traditional risk factors explain less of the cancer risk in Hispanics than in NHW for certain cancers. These unique patterns warrant further investigation to elucidate the effects of treatment, genetics, environment, and lifestyle on cancer risk in this population. In conclusion, studying Hispanic/Latino populations in cancer research is essential to address health disparities, improve cancer prevention and early detection strategies, and develop culturally appropriate interventions. The genetic admixture of this population offers unique opportunities to identify novel risk factors and advance our understanding of cancer etiology [19,20].

This study aims to characterize transcriptomic alterations driving clonal resistance to NAC in BC. By integrating sequencing data with publicly available RNA-seq datasets and advanced computational analyses, we seek to identify key genes implicated in therapy resistance and explore potential drug repurposing strategies. Deciphering these mechanisms will enhance our understanding of NAC resistance and enable identification of novel biomarkers, ultimately guiding development of more precise and effective therapeutic interventions for patients who do not achieve a complete pathological response.

## Materials and methods

### Patient population and samples

Female patients diagnosed with (a) locally advanced mammary adenocarcinoma (stage IIB–IIIC) at the National Cancer Institute of Colombia (INC) between September 2013 and March 2021 and (b) candidates for NAC were enrolled in this study. All participants provided written informed consent through an IRB-approved (Institutional Review Board) consent form, in which they were informed of the use of their samples (formalin-fixed paraffin-embedded or FFPE) and medical records for research purposes. This study was approved by the Comité de Ética e Investigaciones (IRB equivalent) of the INC (Protocol C1901300403). This study was conducted in accordance with the principles of the Declaration of Helsinki. Data used in this retrospective study were accessed for research purposes between April 1^st^ 2019 and September 30^th^ 2023. Personal information was anonymized. All data were handled in accordance with institutional and ethical guidelines to ensure participant confidentiality. Most cases included paired pre- and post-treatment samples, except for eight cases in which only sample was available.

### Sample breast cancer collection

To explore gene expression profiles associated with NAC resistance, a set of 58 FFPE samples from non-responders was selected. A pathologist evaluated tumor cellularity for each sample, and only those with ≥50% tumor cellularity from hematoxylin-eosin stained pre-treatment (biopsy) and post-treatment (surgical resection) samples were included in the study. Non-response to NAC was defined based on post-chemotherapy pathology reports, where the presence of invasive tumor cells in both the breast and lymph nodes (ypT0/is ypN0) indicated lack of response. Responders, on the other hand, were those who achieved pathological complete response (pCR), characterized by the absence of invasive tumor cells in both the breast and lymph nodes. Four-micron sections were obtained from the FFPE blocks and manually microdissected.

### RNA extraction

FFPE samples were deparaffinized with two rounds of 1 ml xylene at 50°C for 3 minutes each, followed by two washes with 100% ethanol. Residual ethanol was removed, and samples were air-dried at room temperature. Total RNA was extracted using the Qiagen All Prep DNA/RNA FFPE Kit (Qiagen, Hilden, Germany), automated with QIAGEN QIAcube and MagMAX® FFPE RNA Ultra Kit (Applied Biosystems®, Foster City, CA, USA), following the manufacturer’s protocol. Extracted nucleic material was frozen at −80ºC. RNA quantity and quality were assessed using a Qubit 3.0 Fluorometer (Life Technologies, CA, USA) and an Agilent 2100 Bioanalyzer (Agilent Technologies). RNA integrity was calculated using the DV200 value with Agilent 2011 Bioanalyzer Expert Software, representing the percentage of RNA fragment ≥200 nucleotides in length.

### Library preparation and sequencing

Genomic libraries were prepared using the Illumina TruSeq RNA Exome Library prep kit, following the manufacturer’s protocol with modifications: RNA input was increased to 300ng, samples with DV200 > 30% were chosen, PCR was reduced to 9 cycles, and hybridization time extended to 16 h. These adjustments enhance library diversity and data quality, crucial for FFPE material, which is highly degradable [21]. Libraries were quantified using the Qubit instrument and DNA HS kit (Invitrogen) and assessed with the Agilent 2100 Bioanalyzer using a DNA 1000 chip (pre-capture) or high-sensitivity DNA chip (post-capture). Library concentrations were normalized to 10 nM and pooled for multiplex sequencing. Pooled libraries were denatured, PhiX control added, clustered, and sequenced in a NextSeq500 High-Output flow cell at 2 x 75 bp. Sequencing quality exceeded 90%, with an average read count per sample of 30,105,535.

### RNA sequencing data processing, gene expression analysis of DEGs

Genome alignment, quality control were analyzed using Partek Flow (Partek, St. Louis, MO, USA). Raw reads were trimmed at both ends using a minimum quality score of 20 and a minimum read length of 25. Contaminants were removed with Bowtie 2 v2.2.5, and reads were aligned to the human genome (hg38) using STAR 2.7.3a. Aligned reads were quantified using RefSeq Transcripts 96, retaining genes with at least five reads in 80% of the samples. Differential expression was analyzed using DEseq2 after normalization with variant stabilizing transformation (VST). Benjamini and Hochberg correction (FDR = 0.05) and fold-change (FC) values of 2 were used to identify differentially expressed genes (DEGs). Gene expression analyses compared responses to neoadjuvant chemotherapy across intrinsic subtypes: Luminal A, LuminalB/Her2-, LuminalB/Her2 + , and TNBC. Library preparation, sequencing, and analysis were performed at the Translational Genomics Core, Department of Interdisciplinary Oncology, Louisiana State University Health Sciences Center, New Orleans, LA.

### Functional enrichment analysis of DEGs

Pathway enrichment analysis was performed to identify pathways in nonresponder patients using the Kyoto Encyclopedia of Genes and Genomes (KEGG) [22], Gene Ontology (GO) [23], and Disease Ontology [24] (DO) with the clusterProfiler [25] R package using DEGs from RNA-Seq data. Benjamini-Hochberg false discovery rate (FDR) <0.05 was chosen as cutoff criterion.

### PPI network construction, hub gene selection

A protein-protein interaction (PPI) network for DEGs was constructed using the Search Tool for the Retrieval of Interacting Genes (STRING, version 12.0) database [26] using “Homo sapiens” as reference and using a medium confidence interaction. Experimental evidence and co-expression were selected as active interaction source parameters and full-string network appearance. Modules of PPI network of DEGs with confidence scores greater than or equal to 0.4 were retrieved and imported to Cytoscape3.10 [27]. Cytohubba plug-in in Cytoscape was used to identify the top 10 hub nodes according to maximal clique centrality (MCC) method with neighbors and shortest paths as additional parameters.

### Estimation of relative cell type abundance

Abundance scores for cell types in the tumor microenvironment were estimated using enrichment (xCell [28], 64 immune and stromal cells) and deconvolution (CIBERSORTx [29], 22 immune cells) methods on normalized RNA-seq data. xCell scores correlated highly with true cell proportions but could not be directly interpreted as cell fractions [28]. This approach assigns enrichment scores across all samples by integrating the single-sample gene set enrichment analysis and deconvolution methods. All sequenced samples were analyzed with xCell and CIBERSORTx to compare stromal and immune scores for nonresponders and responders using the non-parametric Mann-Whitney U test with FDR correction for multiple comparisons.

### Integrating Bootstrap and Jackknife techniques to address small sample sizes

To address the challenges associated with small sample sizes and improve result reproducibility, sensitivity analyses were conducted using bootstrap resampling and jackknife methods. Specifically, 20 bootstrap iterations were performed for each molecular subtype using a sampling scheme consisting of 10 pre-NAC and 10 post-NAC samples randomly selected with replacement. This approach enabled the evaluation of consistency across the molecular subtypes. Differential gene expression analysis was performed in each iteration to identify the top 20 differentially expressed genes consistently observed across the bootstrap samples. To assess the variability and consistency of gene expression, several statistical measures were calculated for each gene. The mean fold change was determined by averaging the fold-change values across all iterations, excluding the missing values. The standard deviation of fold changes was computed to quantify variability in gene expression, and the coefficient of variation was derived by dividing the standard deviation by the absolute value of the mean fold change, providing a relative measure of variability. The number of valid data points for each gene was counted by tallying the non-missing fold-change values, ensuring that missing data were appropriately accounted for. Additionally, the jackknife method was applied to evaluate the bias and variance of the estimates, thereby enhancing the generalizability of the findings [30–33]. The combination of these methods allowed for a robust assessment of gene expression stability across assays. Boxplots and heatmaps were generated to assess the consistency of fold changes, identify genes with stable expression patterns, and minimize the impact of small sample sizes.

### Gene expression validation

To validate gene expression findings, publicly available mRNA expression datasets were retrieved from the Gene Expression Omnibus (GEO) database. The keywords “neoadjuvant chemotherapy AND breast cancer AND non-responder” were used in the search, and the results were limited to Homo sapiens as the organism. The inclusion criteria for the search were (1) at least 30 gene expression profiles present; (2) data obtained for the same cancer type and using the same experimental platform; (3) every profile linked with the patient’s clinical history; (4) all cancers treated with at least one common drug or chemotherapy regimen; and (5) available treatment outcomes enabling the classification of every case as either responder or non-responder. Comparative analyses of the pre-NAC and post-NAC samples were performed using the GEO2R web tool. *P* < 0.05, adjusted to FDR, was chosen as the cut-off criterion.

## Results

### Clinicopathologic characteristics of BC patients with incomplete response to NAC

**Fig 1:**
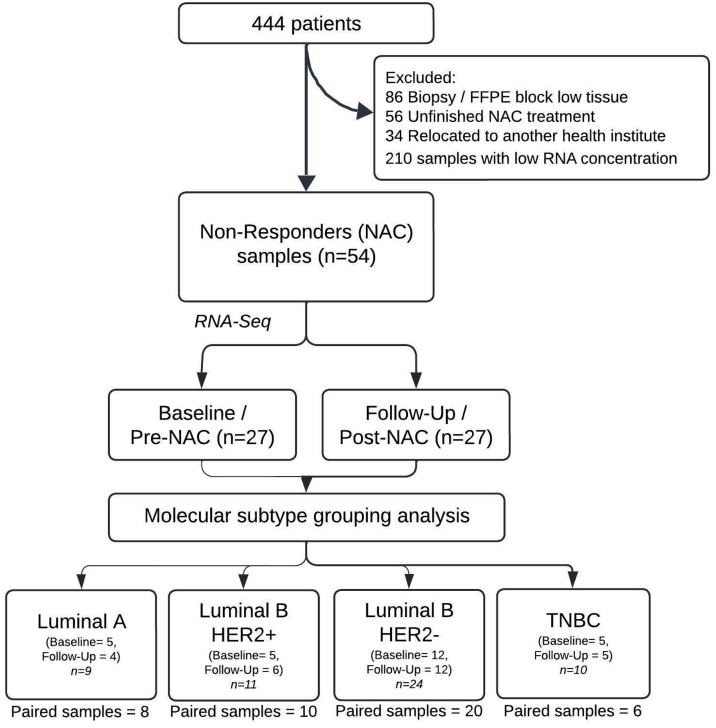
Flow diagram of patient inclusion and molecular subtype groups. Patients were classified as non-responders if they did not achieve pathological complete response (pCR).

Of the 444 patients with locally advanced tumors (Fig 1), 176 were excluded due to incomplete NAC treatment, relocation, or insufficient tissue in their FFPE blocks for RNA extraction. RNA was extracted from FFPE samples of 268 patients who completed the NAC regimen; however, 210 samples did not meet the required minimum RNA concentration and DV200 quality standards necessary for the RNA-seq library preparation. A total of 32 patients who achieved an incomplete pathological response following NAC were included. Table 1 details the clinicopathological characteristics. The median patient age was 53 years. Most patients were overweight or obese, and postmenopausal. The majority were classified as grade II or III, with clinical stage IIIB being the most common. Most patients did not present metastatic nodules or lymphovascular or perineural invasion. Most patients received standard neoadjuvant chemotherapy of anthracyclines and taxanes.

**Table 1:** Demographic and clinicopathological characteristics of the patients with breast cancer.

Variable	N = 32^*1*^
**Age**	53 (45, 63)
**BMI**	27 (25, 30)
**Menopause Status**	
Pre-menopause	5 (16%)
Post-menopause	27 (84%)
**Subtype**	
Luminal A	6 (19%)
LumBHer2-	14 (44%)
LumBHer2+	5 (16%)
TNBC	7 (22%)
**Histological Grade**	
I	1 (3.1%)
II	14 (44%)
III	17 (53%)
**Clinical Stage**	
IIB	5 (16%)
IIIA	4 (13%)
IIIB	15 (47%)
IIIC	8 (25%)
**Lymphovascular Invasion**	
Absent	15 (47%)
Present	6 (19%)
No data	11 (34%)
**Perineural Invasion**	
Absent	7 (22%)
Present	3 (9.4%)
No data	22 (69%)
**Lymph node metastasis**	
M0	32 (100%)
**Treatment**	
Standard (AC + T)	20 (63%)
With Carboplatin	5 (16%)
With HER2 blockade	6 (19%)
With Taxanes	1 (3.1%)
**Status**	
Alive	15 (47%)
Deceased	17 (53%)

Data are presented as numbers (percentages), unless otherwise indicated.

^*1*^ Median (Q1, Q3); n (%), AC = Anthracycline, T=Taxanes

### Differences in transcriptome profiles before and after NAC treatment

**Fig 2:**
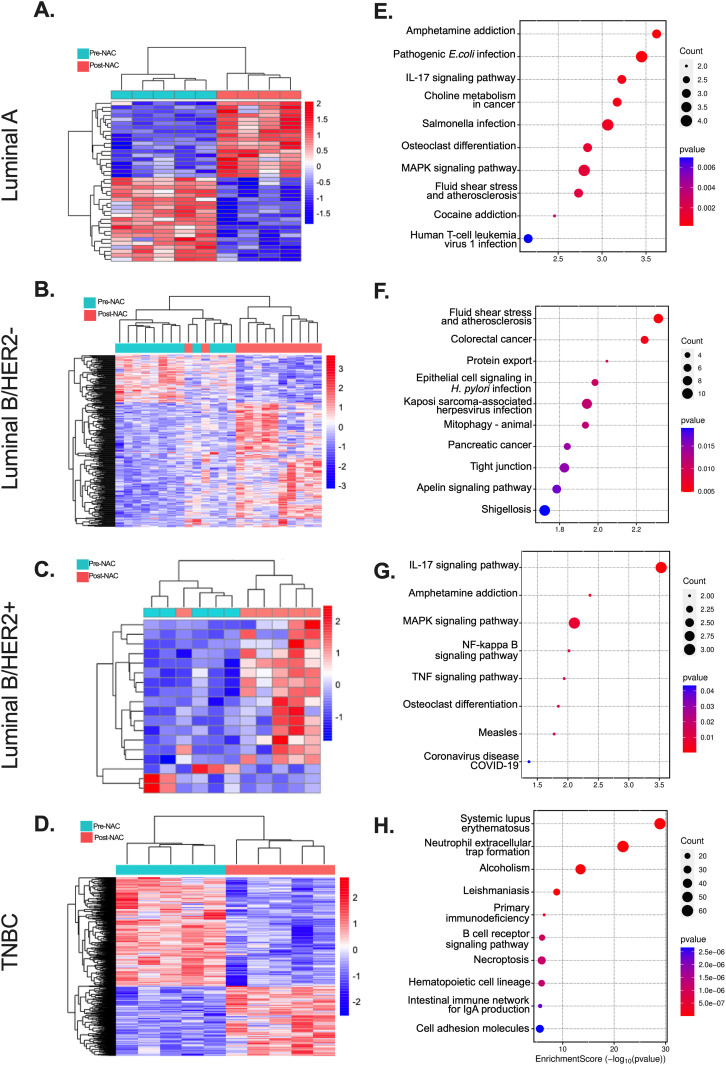
Unsupervised hierarchical clustering and functional Analysis of RNAs in Non-responder patients to NAC showing DEGs predominantly segregated pre- and post-NAC samples among (A) Luminal A (n = 40 DEGs), (B) LuminalB/HER2+ (n = 18 DEGs), (C) LuminalB/HER2- (n = 342 DEGs), and (D) TNBC (n = 1385 DEGs). Functional analysis (significantly enriched pathways) of differentially expressed genes in non-responder samples post-NAC and pre-NAC in Luminal A (E), LuminalB/HER2- (F), LuminalB/HER2+ (G), and TNBC (H) subtypes.

To identify mRNA biomarkers associated to NAC non-responsiveness, RNA-Seq was used for transcriptome profiling. Unsupervised clustering analysis revealed distinct gene expression patterns between pre- and post-NAC samples across the different molecular subtypes (Fig 2A–D). Differential expression analysis using DESeq2 identified DEGs in post-NAC samples (S1 Table). Unsupervised hierarchical clustering showed that DEGs predominantly segregated pre- and post-NAC samples among Luminal A (40 DEGs, Fig 2A), LuminalB/HER2+ (18 DEGs, Fig 2B), LuminalB/HER2- (342 DEGs, Fig 2C), and TNBC (1385 DEGs, Fig 2D), highlighting the heterogeneous transcriptomic profile among different breast cancer subtypes.

### Enriched pathways associated with nonresponse to NAC

**Fig 3:**
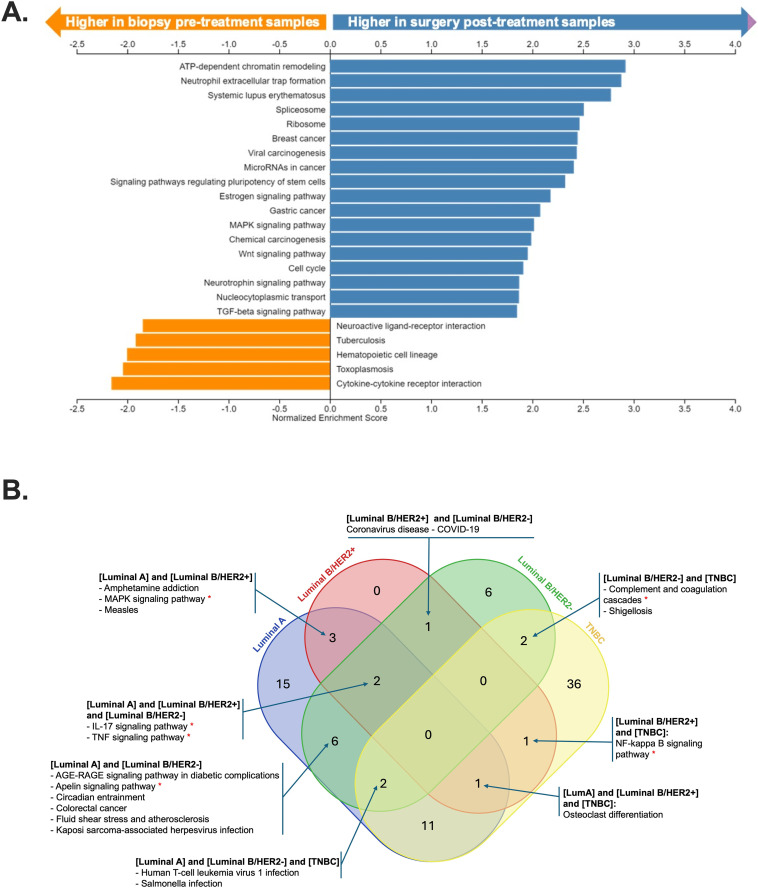
Gene set enrichment analysis (GSEA) analysis across all subtypes (A). Venn diagram depicting shared KEGG enriched pathways (B).

Gene ontology and KEGG pathway enrichment analyses were conducted on DEGs to identify biological processes, cellular components, and molecular functions (S1A–D Fig), in addition to significantly enriched pathways (Fig 2E–H) on pre- and post-NAC across molecular subtypes. The top 10 enriched biological processes from clusterProfiler analysis under Gene Ontology terms are shown in the dot plot for various comparison groups (Fig 2E–H). Gene set enrichment analysis (GSEA) analysis across all subtypes revealed an upregulation of neutrophil extracellular trap formation, microRNAs in cancer, stem cell pluripotency signaling, and MAPK and Wnt pathways in post-NAC, whereas cytokine-cytokine receptor interaction was highe r pre-NAC (Fig 3A). Key shared enriched terms included apelin, IL-17, MAPK, NF-kappa B, TNF signaling pathways, complement and coagulation cascades (Fig 3B), indicating NAC’s influence on mRNAs for proteins in these pathways.

### Integration of DEGS to identify key signaling pathways

To elucidate signaling pathways associated with NAC resistance, DEGs were integrated into MSigDB hallmark gene set. Comparisons were performed with annotated gene sets associated to cisplatin, docetaxel, and doxorubicin resistance as well as angiogenesis, antigen processing, apoptosis, DNA repair, EMT, interleukin-2, PI3K-AKT-MTOR, WNT beta-catenin, Notch signaling, TNFA, TGF-β, and Hedgehog signaling, and identified several genes shared with our samples across subtypes (Table 2). These shared genes were further classified within gene families such as cytokines, growth factors, transcription factors, cell differentiation markers, protein kinases, translocated cancer genes, oncogenes, and tumor suppressors (Table 3). This classification provides insights into the molecular mechanisms underlying NAC resistance and highlights potential targets for therapeutic intervention.

**Table 2:** Hallmark Gene set collection comparison between DEGs and Molecular Signatures Database (MSigDB) across molecular BC subtypes.

Pathways	Luminal A	Luminal B/HER2+	Luminal B/HER2-	TNBC
Angiogenesis				*CCND2, CXCL6, VEGFA*
Antigen processing and presentation			*CANX, NFYB*	*CIITA, HLA-DMA, HLA-DMB, HLA-DOA, HLA-DOB, HLA-DPB1, HLA-DRB5, HLA-E, HLA-F, RFXANK, TAP1*
Apoptosis	*ATF3, JUN*		*ATF3, CCNA1, CD69, GADD45B, IGFBP6, JUN, NEFH, PEA15, TXNIP*	*ATF3, BGN, BIRC3, CASP4, CCND2, CFLAR, DPYD, F2, GUCY2D,HMGB2, IL1B, IRF1, JUN, MADD, PLCB2, RNASEL, TAP1*
Cisplatin_DN		*CHRDL1*		*ADM, BAMBI, IDO1, MMP10, PBDC1, PCDH7, SKAP1*
Cisplatin_UP				*PEG10, TFPI2*
Complement		*TNFAIP3*	*ANXA5, SERPINE1*	*APOBEC3G, CASP10, CASP4, CD40LG, CP, CR1, CR2, DOCK10, DPP4, ERAP2, F10,F2, IRF1, ITGAM, JAK2, LCP2, LTF, PIK3 CG, PIK3R5, PSMB9, TFPI2, WAS, ZFPM2*
DNA repair			*CETN2, COX17, DGCR8, POLR2K, RALA*	*FEN1, HPRT1, NCBP2, NUDT21, POLR2F, POLR2K, SSRP1, TAF10, TAF12*
Docetaxel resistance			*ATP5F1E, DYNLL1*	*FXR1, GAPDH,GSTP1,PDCD5,RPL38,S100A10*
Doxorubicin			*ABCB1, CMPK1*	*GJA5, PSG4, TYMP*
Doxorubicin_up	*FOSB,POLQ*	*FOSB*	*FOSB*	*BIRC5, CDC20, CDCA8, CDKN3, HMGB2, HMMR, KIF4A, MAD2L1,MKI67, NUSAP1, PBK, PCLAF, TPX2*
EMT	*GEM, JUN*	*TNFAIP3*	*ELN, FBLN1, GADD45B, GEM, JUN, PTX3, SERPINE1, SERPINE2, SFRP4*	*ADAM12, BGN, CXCL12, CXCL6, FBN2, FGF2, GREM1, IGFBP2, IGFBP3, IL15, JUN, MGP, PDLIM4, PTX3, SERPINE2, TFPI2, TGM2, VEGFA*
Fatty acid			*GABARAPL1*	*ACSS1, ADH1C, BMPR1B, CCDC58, HADH, HSD17B11, MIF, PDHA1, S100A10, SMS, XIST*
Hedgehog_signaling				*HEY1, VEGFA*
IFNa			*TRIM25, TXNIP*	*GBP4, IL15, IL7, IRF1, LPAR6, PSMA3, PSMB8, PSMB9, STAT2, TAP1, TRIM25, UBA7*
IL2_STAT5		*ABCB1*	*GABARAPL1, GADD45B, KLF6, NFIL3, SNX9, SWAP70*	*ADAM19, BHLHE40, CAPN3, CCND2, CCR4, GBP4, IL10RA, IL1R2, IL2RB, IRF4, IRF8, ITGAE, MYC, PRKCH, RHOH, S100A1, SCN9A, TGM2, TLR7, XBP1*
IL6_JAK_STAT3	*JUN*		*JUN*	*CD9, CSF2RB, CXCL9, IL12RB1, IL1B, IL1R2, IL6ST, IL7, IRF1, ITGA4, JUN, PIK3R5, STAT2*
Inflammatory response			*CD69, EDN1, HBEGF, KLF6, NAMPT, RGS1, SERPINE1*	*ABCA1, ADM, CCR7, CD40, CXCL6, CXCL9, IL10RA, IL15, IL1B, IL2RB, IRF1, KCNA3, LCP2, MYC, PCDH7, PDPN, PIK3R5, PTGER4, RGS1, SLAMF1*
Mtor signaling pathway (KEGG)			*CAB39, IGF1*	*DDIT4, EIF4E, PIK3 CG, PIK3R5, RHEB, TSC1, VEGFA*
Notch_signaling				*HES1, WNT2*
PI3K-AKT-MTOR			*CAB39, RAC1, RALB, UBE2N*	*ARF1, EIF4E, PRKCB, SLC2A1, STAT2*
Protein secretion			*RAB2A, STX12, STX7*	*ABCA1, ANP32E, ARF1, LMAN1, MON2, RAB2A, TPD52*
Response_to_interleukin_17 (GOBP)				*IL1B*
TGF_BETA_signaling			*JUNB, PPP1R15A, SERPINE1, WWTR1*	*LEFTY2, RAB31*
TNFA_Signaling_VIA_NFKB	*ATF3, DUSP1, EGR1, FOS, FOSB, GEM, JUN, NR4A1*	*DUSP2, FOS, FOSB, NR4A1, TNFAIP3*	*ATF3, CD69, CXCL2, CCN1, DUSP1, EDN1, EGR1, EGR2, FOS, FOSB, GADD45B, GEM, HBEGF, JUN, JUNB, KLF2, KLF4, KLF6, KLF9, NAMPT, NFIL3, NR4A1, NR4A3, PER1, PPP1R15A, PTGS2, PTX3, RCAN1, SERPINE1, ZFP36*	*ABCA1, ATF3, BHLHE40, BIRC3, CFLAR, CXCL6, DRAM1, DUSP1, EFNA1, EGR1, FOS, HES1, IL1B, IL6ST, IRF1, JUN, MYC, NR4A1, NR4A2, PER1, PTGER4, PTX3, TAP1, VEGFA*
Wnt_beta_catenin_signaling.				*CCND2, GNAI1, HEY1, MYC*

**Table 3:** Classification of shared genes into gene families of shared genes by molecular subtypes and annotated gene lists in The Molecular Signatures Database (MSigDB) databases.

Gene Families	Cytokines and growth factors	Transcription factors	Cell differentiation markers	Protein kinases	Translocated cancer genes	Oncogenes	Tumor supressors
Angiogenesis	*CXCL6, VEGFA*				*CCND2*	*CCND2*	
Antigen Processing and Presentation (KEGG)		*CIITA, NFYB, RFXANK*			*CIITA*	*CIITA*	
Apoptosis		*ATF3, HMGB2, IRF1,JUN*		*GUCY2D,RNASEL*	*CCND2*	*CCND2, JUN*	
Complement	*CD40LG*	*IRF1,ZFPM2*	*CD40LG, CR1,CR2,DPP4,ITGAM*		*JAK2*	*JAK2*	*TNFAIP3*
DNA repair		*SSRP1,TAF10,*					
Docetaxel Resistance							
Doxorubicin Resistance	*TYMP*		*ABCB1*				
Doxorubicin resistance (overexpressed genes)		*HMGB2, FOSB*	*HMMR*	*PBK*			
EMT	*CXCL6,CSCL12,FGF2, GREM1, IL15, VEGFA*	*JUN*			*ELN*	*ELN,JUN*	*TNFAIP3*
Fatty Acid metabolism	*MIF*		*BMPR1B*	*BMPR1B*			
Hedgehog Signaling	*VEGFA*	*HEY1*					
IFNa	*IL7, IL15*	*IRF1, STAT2,TRIM25*					
IL2 STAT5		*BHLHE40,IRF4,IRF8,MYC,NFIL3,XBP1*	*ABCB1,CCR4,IL10RA,IL1R2, IL2RB, ITGAE,*	*PRKCH*	*CCND2, IRF4, MYC, RHOH*	*CCND2, IRF4,MYC, RHOH*	*IRF4,KLF6,RHOH,*
IL6 JAK-STAT3	*CXCL9, IL1B, IL6ST*	*IRF1, JUN*	*IL6ST*			*IL6ST, JUN*	
Inflammatory_response	*ADM, CXCL6, CXCL9, EDN1, IL15, IL1B, NAMPT*	*IRF1, KLF6, MYC*	*CD69, CD40, IL10RA, SLAMF1*		*MYC*	*MYC*	*KLF6*
MTOR signaling pathway (KEGG)	*IGF1, VEGFA*						*TSC1*
NOTCH signaling							
PI3K-AKT-MTOR		*STAT2*					
Protein secretion							
Response to Interleukin 17(GOBP)	*IL1B*						
TGF Beta signaling	*LEFTY2*	*JUNB*					
TNFA signaling via NFKB	*CXCL6, CXCL2, EDN1, HBEGF, IL1B, IL6ST, NAMPT, VEGFA*	*ATF3, BHLHE40, EGR1, FOS, FOSB, HES1, IRF1, JUNB, KLF2, KLF4, KLF6, KLF9, NFIL3, NR4A1, NR4A2, NR4A3, PER1, ZFP36*	*CD69, IL6ST*		*BIRC3, MYC, NR4A3, PER1*	*BIRC3, IL6ST, JUN, MYC,NR4A3, PER1*	*KLF6, TNFAIP3*
WNT Beta Catenin signaling		*HEY1*			*CCND2,MYC*	*CCND2,MYC*	

### Intersection of DEGs across molecular subtypes

**Fig 4:**
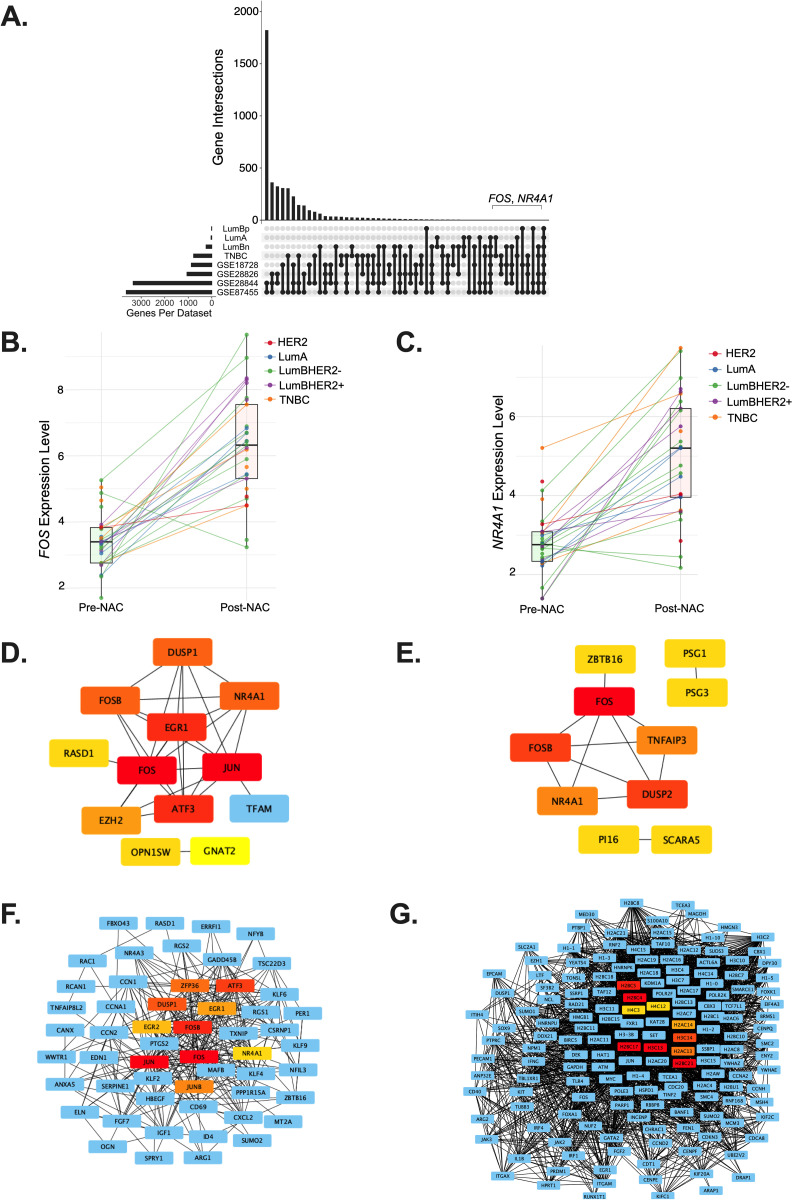
Gene expression analysis and network centrality. (A) Upset plot showing the number of unique and intersecting genes among different datasets. The black bars represent the number of genes in each intersection, and the connected dots indicate which datasets are included in that intersection. All sample sets have *FOS* and *NR4A1* genes in common. (B) and (C) Paired boxplots of *FOS* and *NR4A1* expression levels (normalized counts) in pre- and post-treatment samples, respectively. The gray lines connect the paired samples. The p-values were calculated using the Wilcoxon signed-rank test. (D-G) Hub genes identified by network analysis using the Cytohubba plug-in, ranked by Maximal Clique Centrality (MCC). The networks correspond to (D) Luminal A, (E) Luminal B/HER2 + , (F) Luminal B/HER2-, and (G) Triple-Negative Breast Cancer (TNBC). The color of the nodes indicates centrality: red for high, orange for high-moderate, yellow for low-moderate, and blue for low.

Intersection analysis, visualized via a upset plot [34] (Fig 4A), identified common DEGs across molecular subtypes. *FOS* and *NR4A1* were present in all subtypes, whereas *ZBTB16* was unique to luminal B. *FOSB* was common in all luminal samples. *ATF3*, *DUSP1*, *EGR1*, and *JUN* were shared among HER2-negative subtypes (Luminal A, Luminal B/HER2-negative, and TNBC). *GEM* was common to luminal A and Luminal B/HER2-negative subtypes. Validation using publicly available geoDatabases (GSE18728, GSE28844, GSE28826, and GSE87455) confirmed *FOS* and *NR4A1* presence across all groups, whereas other DEGs were shared among subtypes. Further comparative analysis of pre- and post-treatment sample changes based on gene expression using the Wilcoxon signed-rank test revealed a significant increase in *FOS* and *NR4A1* expression levels in post-treatment tumor samples (p < 0.01, Fig 4B–C). These findings suggest a potential role of these genes in NAC resistance across different BC subtypes. The top 10 nodes (from PPI network analysis) ranked by MCC method were chosen: *FOS*, *NR4A1,* and *FOSB* were hubs found in Luminal A, Luminal B/HER2+ and Luminal B/HER2- analyses. A set of histone genes were among the top 10 hub nodes in TNBC (S2 Table). Luminal A network (Fig 4D) had 12 nodes and 28 edges. Luminal B/HER2-positive network had 10 nodes and 12 edges (Fig 4E). Luminal B/Her2-negative network (Fig 4F) had 54 nodes and 311 edges, whereas TNBC network had 162 nodes and 3038 edges (Fig 4G).

### Tumor microenvironment (TME) infiltration estimation

**Fig 5:**
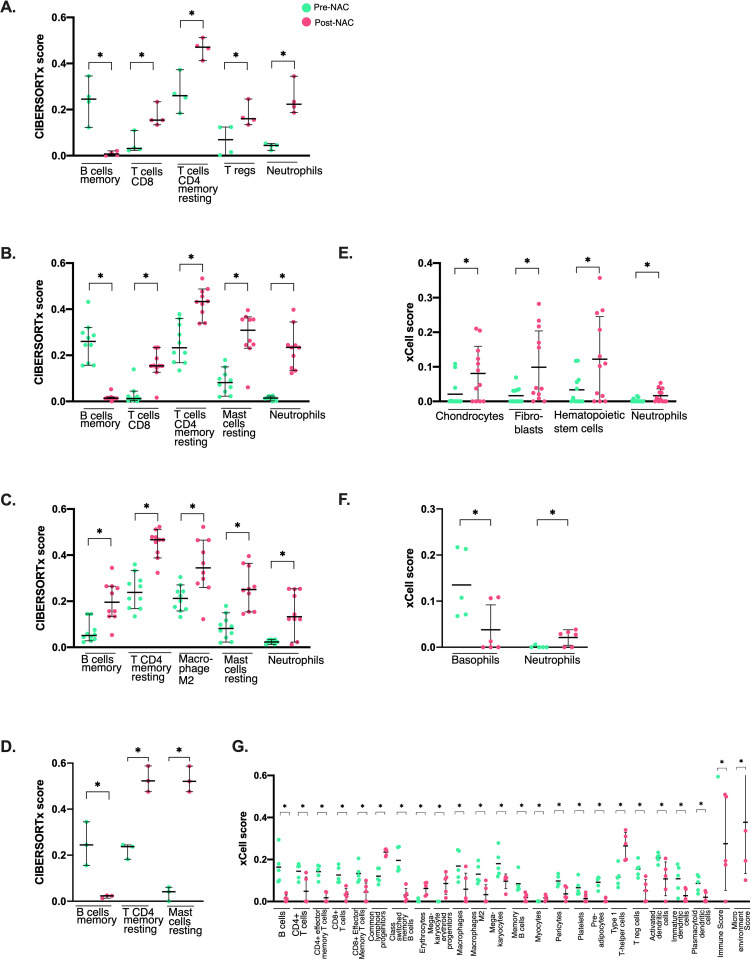
Cellular heterogeneity landscape of tissue expression profiles among different molecular subgroups using the CIBERSORTx(D, H, L, P, T) and xCell (C, G, K, O, S) approaches. Luminal A(A), LuminalB/HER2+(B,E), LuminalB/HER2-(C,F), and TNBC (D,G).P < 0.05; as determined by non-parametric U-Mann Whitney between pre-NAC (light green) and post-NAC (pink) samples. No significant differences were found in xCell scores between Luminal A pre-NAC and post-NAC samples.

CIBERSORTx TME analysis provided insights into immune cell composition before and after NAC across molecular subtypes. In Luminal A post-NAC samples, CIBERSORTx revealed elevated levels of CD4 memory resting T cells, CD8 T cells, regulatory T cells (Tregs), and neutrophils, while xCell showed no statistical differences. For Luminal B/HER2 + post-treatment, CIBERSORTx identified increased CD8 T cells, CD4 memory resting T cells, resting mast cells, and neutrophils, with xCell also showing higher neutrophil fractions. In Luminal B/HER2negative, CIBERSORTx detected higher levels of B cell memory, CD4 T memory resting, Macrophages M2, resting mast cells, and neutrophils in post-NAC, corroborated by xCell’s findings of increased neutrophils. In TNBC post-samples, CIBERSORTx showed elevated resting mast cells and memory CD4 resting T cells along with higher levels of common lymphoid and megakaryocyte-erythroid progenitors and type 1 T-helper cells (Fig 5). Overall, memory B cells increased in pre-NAC samples for all subtypes. CD4 memory resting T cells increased in post-NAC samples in all subtypes. CD8 T cells were increased in Luminal A and Luminal B/HER2 + post-NAC samples. Neutrophils were increased in post-NAC samples in Luminal subtypes. Resting mast cells increased in post-NAC in Luminal B/HER2 + , Luminal B/HER2- and TNBC. Mean xCell and CIBERSORTx scores, along with p-values for all TME cell types, are provided in S3 and S4 Tables, respectively. These results highlight the immune landscape alterations associated with NAC response across subtypes.

### Bootstrap Analysis of Gene Expression Variability across molecular subtypes

To assess the reproducibility of gene expression across molecular subtypes, a bootstrap resampling approach was employed using a sampling scheme of 10 pre-NAC and 10 post-NAC samples for each molecular subtype. S2 Fig analyzes Luminal A and Luminal B/HER2- subtypes, while S3 Fig focuses on Luminal B/HER2+ and TNBC. The bar plots (Panels A,D) illustrate the fold-change variability (standard deviation, SD) across 20 bootstrap iterations for the top 20 DEGs. A lower SD indicates a more consistent fold change across the iterations, suggesting a more reliable result. The box plots (Panels B,E) visualize the distribution and direction (upregulated or downregulated) of the log2 fold changes for each gene. The heatmaps (Panel 1C, F) provide a visual summary of the gene expression patterns across all bootstrap iterations, with red indicating upregulation and blue indicating downregulation. The analysis highlights specific findings for the *FOS* and *NR4A1* genes, demonstrating their subtype-specific expression patterns. *FOS* show a consistent and strong upregulation particularly in Luminal B/HER2- and TNBC, with a tight distribution of fold changes in Luminal A and LuminalB/HER2 + . Similarly, the *NR4A1* gene exhibits distinct, reliable expression patterns. It is consistently upregulated particularly in Luminal B/HER2- and TNBC, with low fold-change variability as shown by their respective bar plots. These results underscore how the expression of key genes like *FOS* and *NR4A1* varies significantly and consistently between different breast cancer molecular subtypes.

## Discussion

Our study identified several DEGs associated with NAC non-responsiveness in BC, notably *FOS* and *NR4A1*, upregulated across all molecular subtypes, which is consistent with the existing literature on BC pathogenesis and treatment resistance.

The *FOS* gene (Fos Proto-Oncogene), particularly c-Fos (*FOS* gene cellular variant), drives chemotherapy resistance in BC by activating survival pathways, regulating membrane biogenesis, and altering gene expression [35]. As part of the AP-1 complex, c-Fos promotes proliferation and survival, enabling resistance to apoptosis. Sustained JNK (c-Jun N-terminal kinase) activation increases c-Fos expression, helping cancer cells withstand chemotherapy stress [36]. In aggressive BC, GATA3 loss shifts gene expression, enhancing epithelial-mesenchymal transition (EMT) and CSC properties [37], further driving resistance. Additionally, FOS boosts radiation resistance and weakens CD8 + T cell anti-tumor activity in TNBC via PI3K/AKT signaling pathway [38].

*NR4A1*, a nuclear receptor family member, is involved in carcinogenesis, angiogenesis, apoptosis, DNA repair, proliferation, metabolism, and inflammation [39]. Its role is complex and context-dependent, influencing cellular processes that impact treatment outcomes. Depending on the tumor type, environment, and stimuli, NR4A1 can promote or inhibit malignancy. *NR4A1* expression is often downregulated in BC tissues, especially in aggressive subtypes like TNBC, correlating with advanced tumor stage, lymph node metastasis, and poor prognosis [40,41]. Restoring *NR4A1* levels in TNBC cell lines inhibits proliferation and invasion, suggesting its tumor suppressor potential [40]. Conversely, *NR4A1* exhibits pro-oncogenic activity in solid tumor-derived cell lines and is frequently overexpressed in lung, pancreatic, and colon tumors. High *NR4A1* expression is a prognostic indicator of decreased survival [42].

High *NR4A1* expression is linked to better survival in BC, suggesting its potential as a NAC response marker [41,43,44]. It regulates apoptosis and cell cycle progression, enhancing therapy sensitivity via ERK signaling pathway [44]. In our study, *NR4A1* upregulation in resistant luminal subtypes aligned with a pro-oncogenic role, yet its loss has been associated with NAC resistance [42,45]. Low *NR4A1* levels reduce apoptosis, promoting cell survival and chemotherapy failure. Its dual role in cancer suggests resistance mechanisms may vary by molecular context, requiring further investigation.

Enriched pathways vary among subtypes, including signal transduction and estrogen biosynthesis. Signal transduction pathways are differentially activated across BC subtypes. MAPK pathway is frequently activated in ER-positive tumors through growth factor stimulation, whereas TNBC often shows alterations in the JNK signaling pathway during treatment resistance [46,47]. The convergence of these pathways often leads to therapeutic resistance, complicating treatment strategies. The identification of specific enriched pathways among BC subtypes underscores the need for personalized treatment strategies that consider the unique biological characteristics of each subtype. This approach can enhance NAC efficacy and improve patient outcomes.

Research has shown that specific immune cell gene expressions correlate with clinical outcomes following NAC. The expression patterns of immune and stromal cells are crucial for determining tumor response to chemotherapy, suggesting the TME’s vital role in mediating these responses [48,49]. The infiltration of immune cell populations following NAC in BC is complex and varies by subtype and pathological response [50]. While some studies indicate beneficial increases in certain immune cells, others reveal shifts towards immunosuppressive environments hindering anti-tumor responses. Understanding these dynamics is crucial for optimizing treatment strategies and improving patient outcomes in BC management. Few studies have systematically characterized chemotherapy’s effect on the tumor immune milieu, particularly during or after treatment in clinical settings.

Our analysis revealed subtype-specific immune alterations in the TME post-NAC. Luminal A showed increased CD4 memory resting T cells, CD8 T cells, Tregs, and neutrophils. Luminal B/HER2 + had elevated CD8 T cells, CD4 memory resting T cells, resting mast cells, and neutrophils, while Luminal B/HER2- exhibited higher B cell memory, CD4 memory resting T cells, M2 macrophages, resting mast cells, and neutrophils. TNBC showed increased resting mast cells and CD4 memory resting T cells. The X-cell score indicated a decline in principal effector cells. These findings highlight NAC’s role in reshaping the TME, potentially favoring chemotherapy-resistant cells promoting tumor invasiveness [51]. Notably, increased Tregs in Luminal A post-NAC may compromise antitumor responses, promoting resistance and metastasis. A concurrent rise in CD8 + T cells and Tregs suggests an imbalance that could weaken immune-mediated tumor control [51].

Neutrophil elevation across subtypes in our study highlights their dual role in cancer. They adapt to the TME, promoting metastasis and suppressing T cells, aiding tumor survival [52]. Some chemotherapy regimens increase neutrophil infiltration, linked to poor outcomes. IL-17 signaling, enriched in Luminal A and Luminal B/HER2 + , drives resistance by recruiting neutrophils and altering the TME [53,54]. Targeting IL-17 pathways may help overcome resistance and improve treatment efficacy.

Characterizing tumor-associated macrophages (TAMs) pre- and post-therapy reveals their role in treatment resistance and metastasis. We observed increased M2 macrophages in Luminal B/HER2- tumors, aligning with reports of elevated CD163 + macrophages and EGFR expression in tamoxifen-resistant ER + /HER2- tumors [55]. A study on paired pre/post-chemotherapy samples highlighted immunosuppressive macrophage expansion in residual disease, reinforcing their role in resistance [56]. Significant M2 infiltration, marked by high CD68 and CD163, correlates with poor prognosis. Our findings show NAC alters the TME immune landscape, underscoring the need to modulate it to enhance therapy efficacy and overcome resistance.

Understanding NAC resistance is crucial for developing targeted therapies in BC. Resistance arises from intrinsic factors like genetic and epigenetic alterations and tumor heterogeneity [57–59]. Intrinsic factors include genetic and epigenetic alterations and tumor heterogeneity. Pre-existing mutations and epigenetic changes drive inherent resistance, while tumor diversity leads to varied treatment responses, complicating efficacy prediction [60].

TME and cancer stem cells drive resistance by supporting tumor survival and drug evasion. TME influences heterogeneity and therapy response through signaling interactions [60,61], while immune cells, fibroblasts, and endothelial cells secrete factors that promote resistance [62]. Hallmark gene set analysis (Table 2) revealed EMT-related genes across all subtypes. EMT, driven by TME signals, enhances drug resistance by increasing cancer cell migration and invasion [13], emphasizing NAC resistance complexity and potential therapeutic targets.

Our findings identified *FOS* and *NR4A1* as potential biomarkers of NAC resistance in BC. *FOS*, which plays a role in chemotherapy resistance through the AP-1 complex and JNK activation, can be utilized as a predictive marker for early signs of treatment failure. Monitoring *FOS* expression could enable early adjustment of treatment regimens, especially in cases of resistance. *NR4A1*, which has both tumor-suppressing and oncogenic roles, is associated with chemotherapy sensitivity and resistance. Its expression level could potentially predict NAC response, particularly in TNBC, where it is often low. These biomarkers could guide personalized treatment strategies, but further clinical validation is required. *FOS* and *NR4A1* also present the possibility of drug repurposing. Targeting FOS-mediated resistance mechanisms, such as JNK/AP-1 signaling, could involve repurposing MAPK or c-Jun inhibitors to enhance the NAC response. Restoration of *NR4A1* function in resistant tumors (e.g., TNBC) or inhibition of its oncogenic function in others could improve treatment response. Examining existing drugs that modulate *FOS* and *NR4A1* could be a quick path to restoring NAC response in BC patients.

Our findings provide valuable insights and directions for future research; however, there are several limitations to consider. First, the sample size and the lack of functional analysis of candidate DEGs limit the depth of our conclusions. Sample size constraints restricted our ability to fully explore the molecular characteristics associated with therapy resistance in this population. Additionally, the study’s analysis of TME relied solely on computational deconvolution algorithms (xCell and CIBERSORTx). As these methods are predictive rather than direct measurements of cellular infiltration, the results should be interpreted with caution. To provide definitive confirmation of the TME composition, future research should employ experimental techniques such as immunohistochemistry or flow cytometry. While this study identifies associations between gene expression changes and TME in relation to NAC resistance, it is crucial to emphasize that these associations do not establish causal relationships. To confirm the mechanistic roles of key hub genes, such as *FOS* and *NR4A1*, and the pathways involved in NAC resistance, future functional studies, including in vitro gene knockdown/overexpression experiments and *in vivo* models, are necessary. While bootstrap and jackknife resampling improved the robustness of our analysis, the small sample size for each molecular subtype limits the generalizability of the findings. We employed 20 bootstrap iterations using a homogenous sampling scheme to evaluate the consistency of our results. However, the potential for bias and reduced precision due to the limited sample size requires caution. The jackknife method helped stabilize the results, but it is also susceptible to small sample size limitations [30,32]. While standard deviation, boxplots, and heatmaps were used to assess fold-change consistency, assay variability and outliers could still influence the outcome. Robust clustering methods, such as those using jackknife distances, can identify co-regulated genes, but assay-specific biases must be carefully considered in any clinical application. Furthermore, our study specifically focuses on a Latin-American cohort, contributing to a more diverse representation in cancer research. However, the absence of comprehensive genetic ancestry data related to treatment response in publicly available cohorts has limited our ability to fully evaluate the ethnic specificity and generalizability of the identified predictive biomarkers. This highlights the significance of our data in addressing this gap. Future studies should incorporate multicenter approaches to validate, strengthen, and broaden our findings, ultimately improving their robustness and applicability across diverse populations and all molecular subtypes. Addressing these limitations in future studies will be essential for translating molecular insights into clinically effective strategies to overcome NAC resistance.

## Conclusion

This study provides valuable insights into the molecular mechanisms underlying NAC resistance in breast cancer, highlighting the role of *FOS* and *NR4A1* in treatment response as well as the enrichment of IL-17, MAPK, NF-kappa B, and TNF signaling pathways. The observed changes in immune cell infiltration patterns across molecular subtypes further emphasize TME influence on chemotherapy resistance. Our findings suggest that NAC modulates gene expression and cellular interactions within the TME, potentially contributing to nonresponsiveness and disease recurrence. A deeper understanding of these mechanisms is essential for refining predictive biomarkers and identifying therapeutic targets. Moving forward, future translational research must focus on developing targeted strategies that can mitigate TME-driven resistance, with an emphasis on clinical application. Such strategies have the potential to significantly improve NAC efficacy, leading to more personalized and effective treatment options for breast cancer patients. Ultimately, the integration of these findings into clinical practice could transform therapeutic approaches and improve patient outcomes in the fight against breast cancer.

## Supporting information

S1 Table Differentially Expressed Genes (DEGs) by BC molecular subtype in post-NAC samples compared with Pre-NAC samples. The table provides the complete list of differentially expressed genes for each subtype, showing the Log_2_Fold Change (Log_2_FC) values.
(XLSX)

S2 Table Top 10 STRING Network Hub Genes Obtained from CytoHubba Analysis. Genes are ranked by the Maximum Clique Centrality (MCC) method, including associated neighbor and shortest path data from the protein-protein interaction network.
(XLSX)

S3 Table xCell Immune and Stromal Cell Type Abundances. Mean abundance values (pre- and post-NAC samples) and p-values from xCell cell types (post-NAC samples compared with Pre-NAC samples) across all four molecular subtypes. Significant values (p < 0.05) are highlighted in bold.
(XLSX)

S4 Table CIBERSORTx Immune Cell Type Abundances. Mean abundance values (pre- and post-NAC samples) and p-values from CIBERSORTx cell types (post-NAC samples compared with Pre-NAC samples) across all four molecular subtypes. Significant values (p < 0.05) are highlighted in bold.
(XLSX)

S1 Fig Gene Ontology and KEGG Pathway Enrichment Analyses. Enrichment analyses for differentially expressed genes are shown with bar plots for biological processes (BP), cellular components (CC), and molecular functions (MF) post-NAC compared to pre-NAC samples across Luminal A (A), LuminalB/HER2- (B), LuminalB/HER2+ (C), and TNBC (D) subtypes.
(TIF)

S2 Fig Bootstrap Analysis of Gene Expression Variability for Luminal A and Luminal B/HER2- Subtypes. (A, B, C) show results for Luminal A (n = 20); (D, E, F) show results for Luminal B/HER2- (n = 20). (A, D) Bar plots of fold-change variability (standard deviation, SD) per gene; (B, E) Box plots of fold changes per gene; (C, F) Heatmaps of gene expression data across assays. The analysis demonstrates the consistency of differential expression patterns.
(TIF)

S3 Fig Bootstrap Analysis of Gene Expression Variability for Luminal B/HER2+ and TNBC Subtypes. (A, B, C) show results for Luminal B/HER2+ (n = 20); (D, E, F) show results for TNBC (n = 20). (A, D) Bar plots of fold-change variability (standard deviation, SD) per gene; (B, E) Box plots of fold changes per gene; (C, F) Heatmaps of gene expression data across assays. The analysis demonstrates the consistency of differential expression patterns.
(TIF)
