# Supplementary figures and images for: Molecular determinants of neoadjuvant chemotherapy resistance in breast cancer: An analysis of gene expression and tumor microenvironment
Source: PLoS One. 2025 Oct 14;20(10):e0334335. doi: 10.1371/journal.pone.0334335 (PMC12520365; doi:10.1371/journal.pone.0334335)

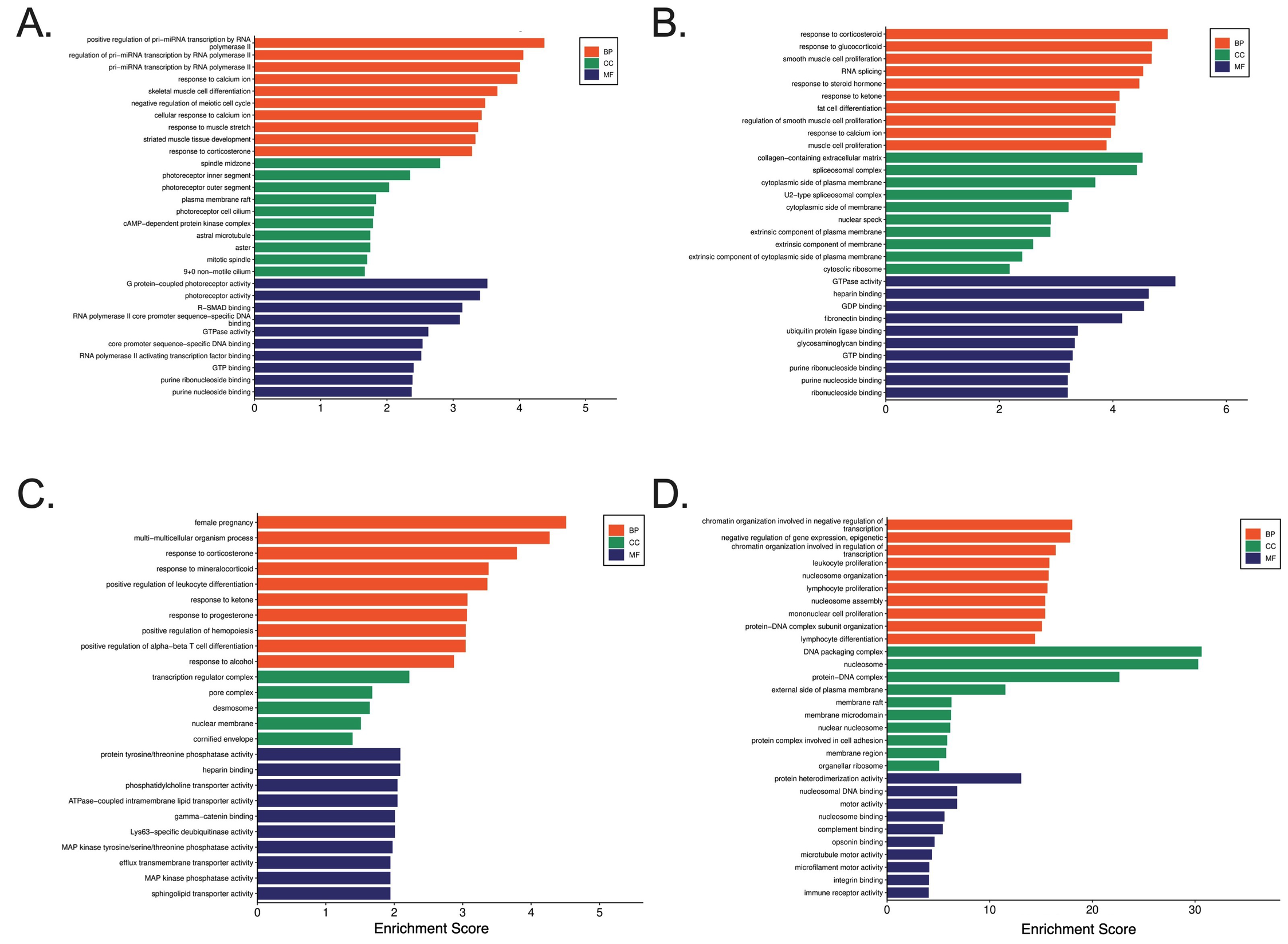

Supplement: S1 Fig — Enrichment analyses for differentially expressed genes are shown with bar plots for biological processes (BP), cellular components (CC), and molecular functions (MF) post-NAC compared to pre-NAC samples across Luminal A (A), LuminalB/HER2- (B), LuminalB/HER2+ (C), and TNBC (D) subtypes. (TIF) [file pone.0334335.s005.tif]

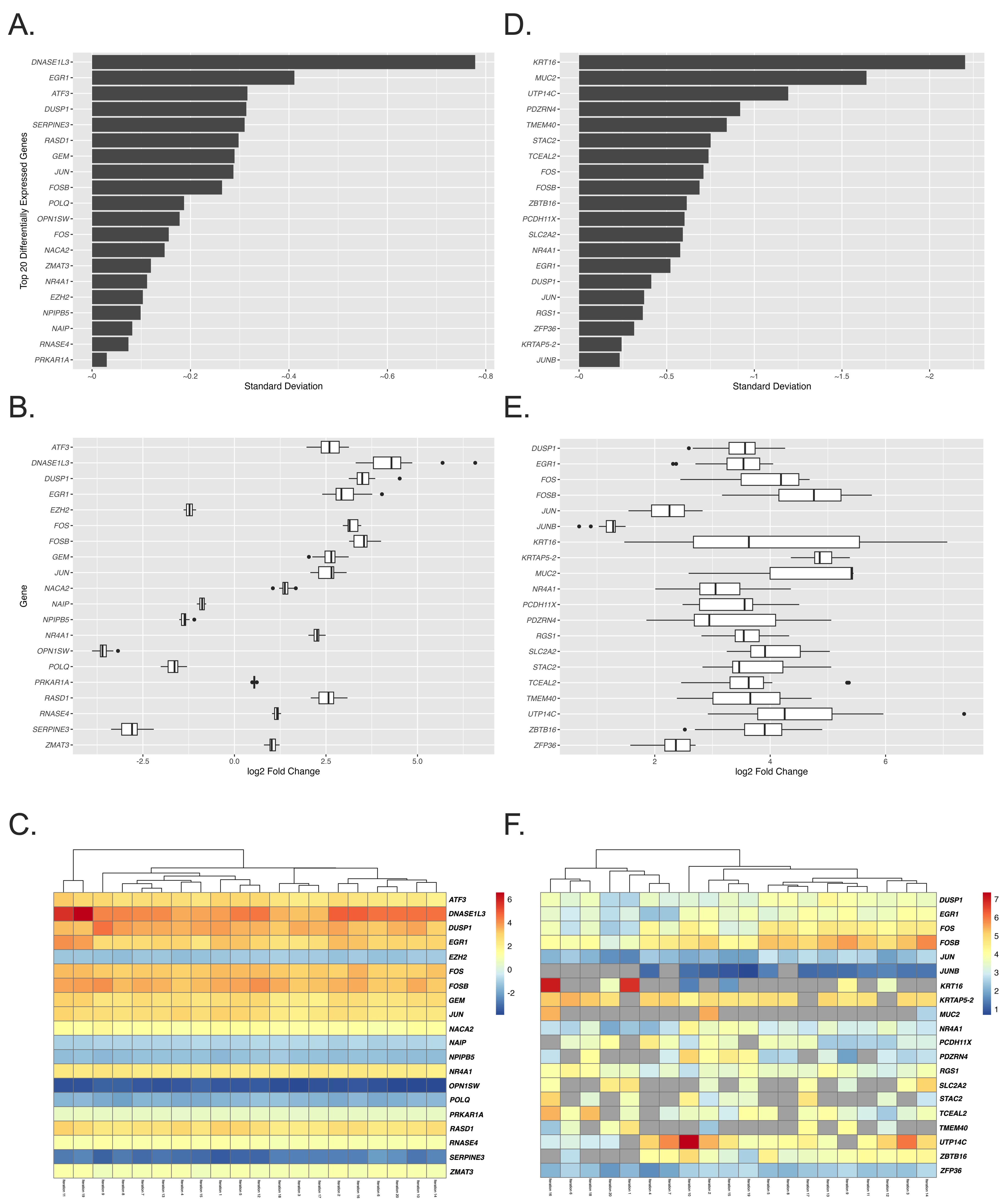

Supplement: S2 Fig — (A, B, C) show results for Luminal A (n = 20); (D, E, F) show results for Luminal B/HER2- (n = 20). (A, D) Bar plots of fold-change variability (standard deviation, SD) per gene; (B, E) Box plots of fold changes per gene; (C, F) Heatmaps of gene expression data across assays. The analysis demonstrates the consistency of differential expression patterns. (TIF) [file pone.0334335.s006.tif]

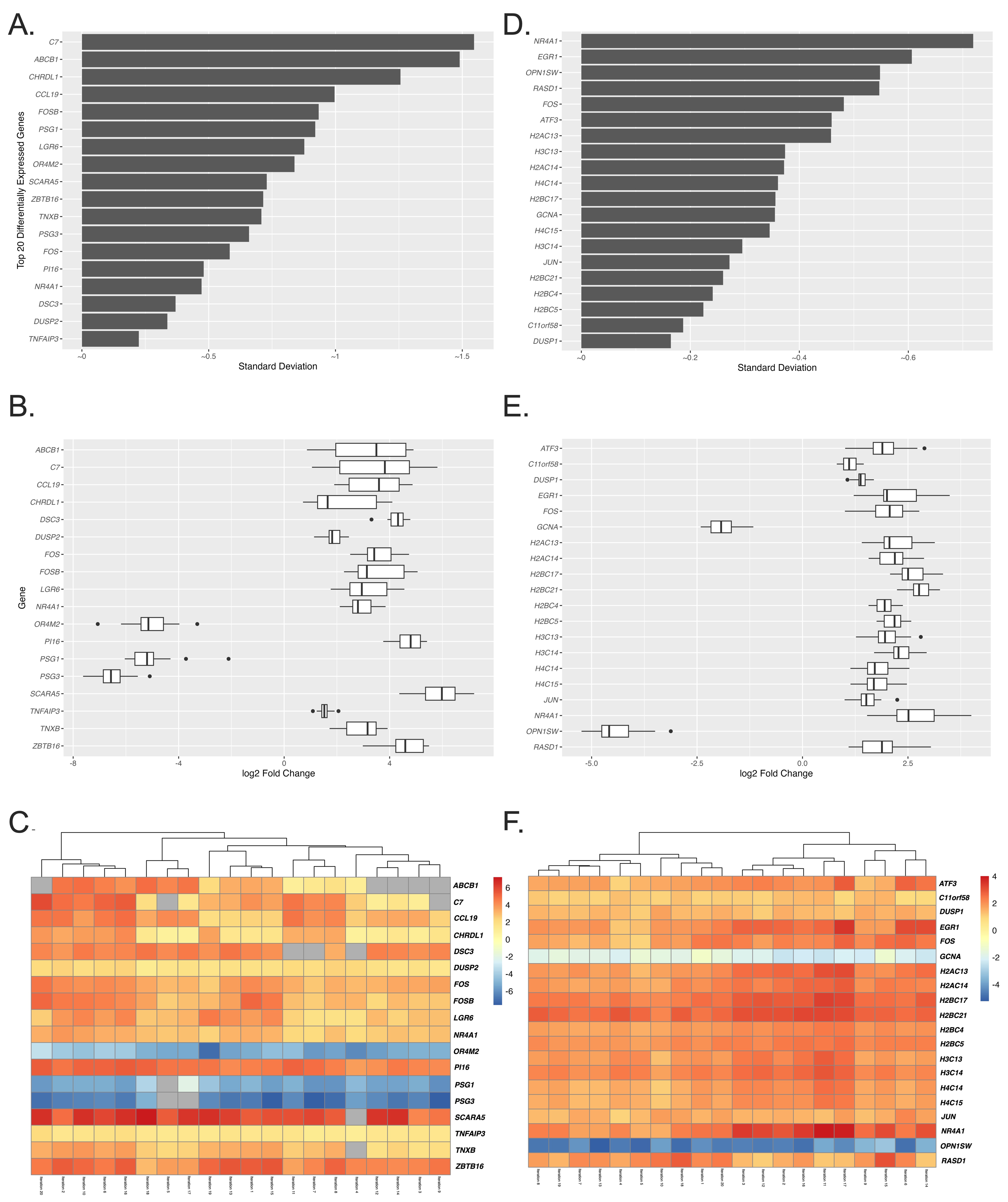

Supplement: S3 Fig — (A, B, C) show results for Luminal B/HER2+ (n = 20); (D, E, F) show results for TNBC (n = 20). (A, D) Bar plots of fold-change variability (standard deviation, SD) per gene; (B, E) Box plots of fold changes per gene; (C, F) Heatmaps of gene expression data across assays. The analysis demonstrates the consistency of differential expression patterns. (TIF) [file pone.0334335.s007.tif]
